# Modeling hydrogen-assisted fatigue crack growth in low-carbon steel focusing on thermally activated hydrogen-dislocation interaction

**DOI:** 10.1080/14686996.2024.2436345

**Published:** 2024-12-20

**Authors:** Osamu Takakuwa, Yuhei Ogawa

**Affiliations:** a Department of Mechanical Engineering, Kyushu University, Fukuoka, Japan; b Research Center for Structural Materials, National Institute for Materials Science (NIMS), Tsukuba, Japan

**Keywords:** Hydrogen embrittlement, fatigue crack growth, temperature dependence, loading rate dependence

## Abstract

Hydrogen-assisted (HA) fatigue crack growth (FCG) occurs in ferritic steels, wherein H-dislocation interaction plays a vital role. We aim to model the HAFCG mechanism based on the *obstruction of dislocations* within the crack tip zone. Our modeling framework is as follows: H is condensed into crack tip and trapped by dislocations; these H significantly decrease dislocation mobility; stress relief via crack blunting is suppressed; localized brittle fracture triggers HAFCG. This model was substantiated experimentally in H_2_ gas at various load frequencies and temperatures. Theoretical formulations were established considering the thermal equilibrium of H-trapping and dislocation breakaway from the H atmosphere.

## IMPACT STATEMENT

Hydrogen-assisted fatigue crack growth was comprehensively interpreted by focusing on thermally activated interaction between hydrogen and dislocation under various temperatures and loading rates.

## GRAPHICAL ABSTRACT

**Figure:**
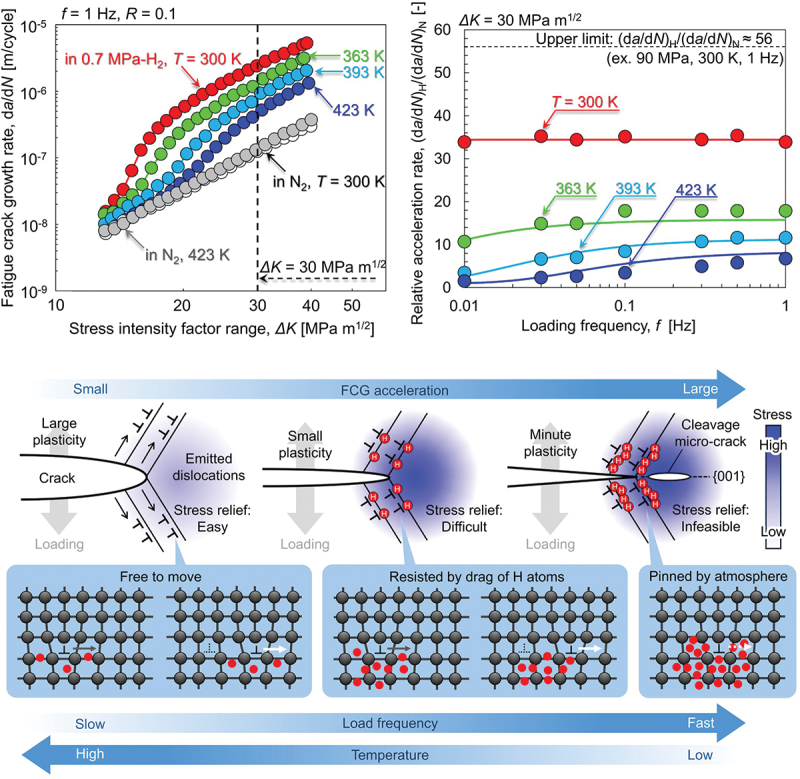

## 1. Introduction

Hydrogen (H) is anticipated to be a potential energy carrier that will contribute to realizing a sustainable and carbon-neutral economy. Hydrogen-related infrastructure is being developed on a global scale. However, since Johnson’s first discovery in 1875 [1], it has been known that H occlusion into structural metallic materials gives rise to degradation of their mechanical properties, such as tensile strength, ductility, fracture toughness, and fatigue life [2–7]. Contact with gaseous H_2_ dramatically accelerates the growth rate of fatigue cracks in a stable Paris regime, a phenomenon often designated as H-assisted (HA) fatigue crack growth (FCG), *i.e*., HAFCG [3,8–13]. This phenomenon manifests in various materials, including ferrite-pearlite steels [8,9,14,15], martensitic steels [13,16,17], austenitic steels [18–20], and nickel alloys [21,22]. The rationales behind such a detrimental event need to be interpreted comprehensively to enable a fracture mechanics-based design to be safely applied to the engineering components utilized in hydrogenated environments. Over the past decades, numerous studies have been carried out to identify the underlying mechanisms responsible for HAFCG, particularly in ferrous materials with a primarily body-centered cubic (BCC) crystal structure [12,23–35]. Nevertheless, the conclusions drawn in these individual studies remain mutually inconsistent due to the complex nature of HAFCG and its dependence on multiple environmental variables, *e.g*., the pressure and temperature of the H_2_ gas environment combined with mechanistic variables such as stress intensity and loading rate.

The models constructed so far for H-assisted fracture, including HAFCG, have been more or less substantiated, albeit indirectly, by experimental evidence, having been extensively discussed in previous studies [12,23–25,28,29,35]. For instance, the segregation of H into the vicinity of the crack tip, driven by stress-assisted diffusion, reduces interatomic bonding and leads to crack propagation preferentially along the cleavage plane (H-enhanced decohesion, HEDE) [12,23]. The presence of H within the crack tip may also locally enhance the mobility of dislocations and increase their velocity, thereby triggering localized ductile fracture (H-enhanced localized plasticity: HELP) [29,36,37]. Furthermore, the adsorption of H atoms on the crack planes facilitates dislocation emission from the crack tip, propelling the crack forward (adsorption-induced dislocation emission, AIDE) [6,38]. Even though these models cannot individually account for the overall behavior of HAFCG, it is likely that several forms of dynamic H-dislocation interaction within the local fracture process zone at the crack tip [12,23,25,29,36] act as key factors in the manifestation of HAFCG. Birenis *et al*. recently revealed that fatigue cracks in pure BCC iron selectively propagate along the {001} cleavage plane in an H_2_ gas environment, accompanied by an unevolved deformation substructure in its crack-wake [12]. Based on this fact, they stated that the presence of H potentially obstructs dislocation motion inside the crack tip zone, maintaining a sharp crack front and thereby making stress relief via crack blunting infeasible. Matsumoto *et al*. performed a long-timescale molecular dynamics simulation of the H-dislocation interaction in BCC iron at various temperatures and dislocation velocities [39]. Their calculation also assumed that, at lower temperatures or faster dislocation velocities, the H-atmosphere around the dislocation would substantially heighten the shear stress required for dislocation motion by exerting drag resistance. These H atoms show repeated short-range jumps close to the dislocation core at higher temperatures and lower velocities, eventually attenuating the effects of obstruction. The responsibility of local pinning such as this for dislocations was similarly demonstrated by Deng and Barnoush in small-scale mechanical tests in an electron microscope [40,41].

To elucidate the nature of H-dislocation interactions that govern HAFCG, one meaningful approach is to focus on the temperature- and loading rate-dependencies of crack growth acceleration, since such interactions are likely to be thermally activatable processes. These dependencies are also of considerable importance for the practical application of engineering steels to various H-related operating conditions. The authors have recently addressed the temperature-dependence of HAFCG in a ferritic-pearlitic low-carbon steel in an H_2_ gas environment, and demonstrated that higher temperatures lead to less HAFCG, the propensity of which also depended on the pressure of H_2_ gas [15,29]. Higher temperatures enhance the thermally-activated desorption of H from dislocations (*i.e.*, de-trapping), deactivating the H-dislocation interaction responsible for HAFCG. A comparison of the deformation substructures beneath the fracture surfaces at 300 K and 423 K showed the dislocations to be less evolved at 300 K where HAFCG was maximized, while evolution was advanced at 423 K due to mitigation of HAFCG. Systematic investigations at various gas pressures and temperatures ranging from 0.7–90 MPa and 300–423 K showed the extent of HAFCG to be successfully described in terms of a single parameter: the ratio of the number of trap sites (dislocations) occupied by H to that of non-occupied trap sites (hereinafter designated as ‘H-occupancy’). Hydrogen-occupancy is a simultaneous function of H_2_ gas pressure and temperature if one assumes that local equilibrium is achieved [42].

Aside from the temperature-effect on HAFCG, a known anomaly was observed when the fatigue loading rate (*i.e*., load frequency) is changed: when the load frequency is reduced at a relatively low gas pressure (*e.g*., 0.7 MPa), crack growth acceleration eventually ceases [24,43,44]. This anomaly has often been interpreted based on plasticity localization models [24,25,36] and the assumption of an H-induced increase in the mobility of dislocations [36,45]. The onset of crack growth acceleration depends on whether H accumulates in concentrated form in the local crack tip zone or exhibits a broader distribution [24]. In short, lowering the load frequency allows H to diffuse more deeply from the crack tip into the material, extending the volume in which dislocation mobility is enhanced, thereby relaxing the localization of plasticity. However, this viewpoint lacks any physical consideration of the thermally activatable character of H-dislocation interactions. If H is trapped by dislocations and obstructs their movement, the dislocation motion that results on overcoming these obstacles may also be a rate-dependent process; and the obstructed dislocations eventually become mobile as the loading rate decreases. By integrating this opposite presumption with the previously-considered H-trapping/de-trapping kinetics, a more consecutive picture can be established based on the full involvement of all three influencing factors: gas pressure, temperature, and load frequency. The present study aims to further elucidate HAFCG with an emphasis on decreasing the mobility of dislocations by H as the primary rationale behind crack growth acceleration. To this end, a series of investigations on the HAFCG in ferrite-based low-carbon steel was carried out in 0.7 MPa H_2_ gas at various temperatures and frequencies, the characteristics of which were modeled through an extensive theoretical formulation.

## 2. Methodology

### 2.1. Materials and experiments

A hot-rolled low-carbon steel (JIS-SM490B) plate with a chemical composition of Fe-0.16C–0.44Si-1.43Mn-0.017P–0.004S (mass%) was examined. Its lower yield stress and ultimate tensile strength measured in ambient air were respectively 360 and 540 MPa. The material possessed a banded ferrite-pearlite microstructure parallel to the rolling direction, with a grain size of approximately 20–40 µm. Compact-tension (CT) specimens with a width *W* of 50.8 mm and a thickness *B* of 10.0 mm were extracted from the longitudinal-transverse (L-T) orientation of the plate. Before the FCG test, a fatigue pre-crack was introduced in laboratory air at room temperature (RT), with a stress intensity factor range Δ*K* of 15 MPa∙m^1/2^, a load ratio *R* of 0.1, and a load frequency *f* of 10 Hz. All the FCG tests were performed according to the ASTM-E647 standard [46] in a 0.7 MPa H_2_ gas or nitrogen (N_2_) gas environment (purity >99.999%) with *R* = 0.1. In the present study, the load frequency *f* and H_2_ gas temperature *T* were varied over a wide range, as *f* = 0.01 ~ 1 Hz at *T* = 300 ~ 423 K. Two types of FCG tests were carried out: a Δ*K-increasing* test with *f* = 1 Hz, acquiring the FCG rate per loading cycle, d*a*/d*N*, under a wide range of Δ*K* at *T* = 300, 363, 393, and 423 K; and a Δ*K-constant* test with Δ*K* = 30 MPa∙m^1/2^, studying the dependencies of FCG rate on *f* and *T* in a fixed mechanical state.

### 2.2. SEM characterizations

The specimens subjected to ∆*K-constant* tests were cut along their mid-thickness parts, and the cross sections of one-half (samples for the characterization of crack paths) were carefully finished by buffing with colloidal SiO_2_ to remove the layer affected by cutting, followed by polishing with coarse slurry. The other half was used for fracture surface observations. The scanning electron microscopy (SEM) characterizations were conducted using a Schottky field-emission SEM (JEOL, IT800) with an acceleration voltage of 15 kV. The crystallography of the crack path was characterized by electron backscattered diffraction (EBSD) on the polished mid-thickness surfaces with beam-step sizes of 300 nm. The acquired orientation maps were utilized to verify local plastic strain distribution through grain reference orientation deviation (GROD) mapping. The GROD value represents the misorientation of each EBSD data point from the average orientation of the grain it belongs to, qualitatively reflecting the density of geometrically necessary (GN) dislocations or intragranular sub-domains divided by GN boundaries created by plastic deformation [47]. More local regions close to the crack tip or crack-wakes were further investigated by electron channeling contrast (ECC) imaging to study the evolutional state of deformation substructures, which can be affected by the emergence of HAFCG.

## 3. Theoretical background to H-dislocation interaction

Our goal is to inclusively interpret HAFCG using various combinations of temperature and load frequency from the perspective of H-dislocation interactions. The main postulation is that H atoms trapped by dislocations behave as obstacles to their motion. To this end, in Section 3, we attempt to formulate the thermal activation kinetics of H-trapping/de-trapping at dislocations. The H-trapping/de-trapping kinetics at a given site obey the McNabb-Foster model [48,49], which expresses the time-dependent partitioning of H from the interstitial lattice site (tetrahedral site) to the trap site. The model provides the transient relationship between trap site H-occupancy, *θ*_T_, and the lattice site occupancy, *θ*_L_, where these site occupancies are defined by the ratio of the number of sites occupied by H among all the sites. The time derivative of trap site H-occupancy, *θ*_T_, is formulated by the difference between the trapping and de-trapping rates with their frequencies *k*_1_ and *k*_2_, when the interstitial lattice site occupancy *θ*_L_ is well below unity,

(1) $$\frac{\partial \theta_{T}}{\partial t}=k_{1}\theta_{L}\left(1-\theta_{T}\right)-k_{2}\theta_{T}$$

(2) $$k_{1}=\nu_{D}\exp\left(-\frac{E_{a}}{\mathrm{RT}}\right)$$

(3) $$k_{2}=\nu_{D}\exp\left(-\frac{E_{a}+E_{b}}{\mathrm{RT}}\right)$$

where *E*_a_ is the activation energy for the lattice diffusion of H (≈6.8 kJ/mol [4,50]), and *E*_b_ is the binding energy of H around the edge dislocation core ( = 47 kJ/mol [51]). *v*_D_ is the Debye frequency (≈10^12^ − 10^13^/s), *R* is the universal gas constant, and *T* is the absolute temperature. The transient of *θ*_T_ over a time duration can be estimated using the following equation.

(4) $$\theta_{T}=\theta_{T,\mathrm{eq}}\left[1-\exp\left(-k_{2}\left\{1+\theta_{L}\exp\left(\frac{E_{b}}{\mathrm{RT}}\right)\right\}t\right)\right]$$

The H-occupancy under an equilibrated condition, *θ*_T,eq_ can then be given by Fermi-Dirac statistics when trapping and de-trapping rates are mutually balanced (*i.e*., ∂*θ*_T_/∂*t* = 0):

(5) $$\theta_{T,\mathrm{eq}}=\frac{\theta_{L}\exp\left(\frac{E_{b}}{\mathrm{RT}}\right)}{1+\theta_{L}\exp\left(\frac{E_{b}}{\mathrm{RT}}\right)}$$

*θ*_L_ is experimentally measured as follows by Quick and Johnson [50], multiplying 1/6, *i.e*., six tetrahedral sites per Fe atom, and $\sqrt{9.86}$ to convert from the atomic ratio of H in bcc Fe to the lattice site occupancy in the former and from the atmospheric pressure to MPa in the latter.:

(6) $$\theta_{L}=9.81\times 10^{-5}\sqrt{F}\exp\left(-\frac{3440}{T}\right)$$

Here, *F* is the fugacity of H_2_ gas, which is determined by the gas pressure, *p*_H_, and the molar volume of H in BCC iron, *V*_M_, as follows.

(7) $$F=p_{H}\exp\left(\frac{V_{M}p_{H}}{\mathrm{RT}}\right)$$

Meanwhile, the local concentration of segregated H around an edge dislocation core, *c*, can be estimated similarly to Equation (5) if one assumes a thermal equilibrium state:

(8) $$c=\frac{c_{0}\exp\left(\frac{E_{b}}{\mathrm{kT}}\right)}{1+c_{0}\exp\left(\frac{E_{b}}{\mathrm{kT}}\right)}$$

(9) $$E_{b}=\frac{\left(1+\nu \right)}{3\pi \left(1-\nu \right)}\mathrm{Gb}\Delta v\frac{y}{x^{2}+y^{2}}$$

where *ν* is Poisson’s ratio, G is shear modulus, and *b* is the Burgers vector. *c*_0_ is the average H concentration in regions distant from the dislocation, given by converting *θ*_L_ to an atomic ratio. Δv is the misfit volume per H atom: 3.57 Å^3^ [52]. The binding energy, *E*_b_, at arbitrary locations around an edge dislocation line, can be calculated using Equation (9) [53]. Here, *x* and *y* denote the distances from the dislocation line along the slip plane and its normal direction, respectively, given that the edge dislocation has its extra half plane oriented along the positive *y*-axis. Even though Equation (8) cannot be applied to the H-segregation in the exact dislocation ‘core’ where linear elasticity breaks down, it still offers a qualitative evaluation of the magnitude of the H-atmosphere around the core at various temperatures.

## 4. Results

### 4.1. Temperature effect on H-trapping/de-trapping

**Figure 1.**
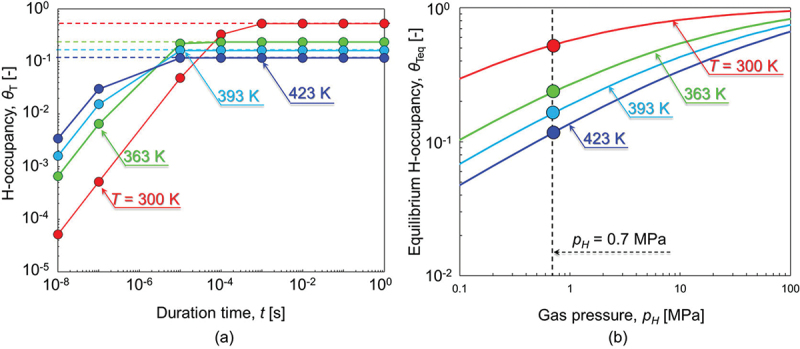
H-trapping/de-trapping state at the dislocation core with the binding energy of 47 kJ/mol for *T* = 300, 363, 393, and 423 K. (a) Time-dependent transient of the H-occupancy at the gas pressure of 0.7 MPa, calculated by Equation (4). The dashed horizontal lines represent the equilibrium H-occupancy at each temperature. (b) H_2_ gas pressure-dependence of the equilibrium H-occupancy calculated by Equation (5).

Figures 1(a,b) showcase the H occupancy, *θ*_T_, and its equilibrium value, *θ*_T,eq_, which are plotted as a function of time duration, *t* (Figure 1(a)) and gas pressure, *p*_H_ (Figure 1(b)), respectively. The calculations were made for *T* = 300, 363, 393, and 423 K with *E*_b_ = 47 kJ/mol [29,51] by using Equation (4) and (5). In Figure 1(a), one can observe that the H-trapping/de-trapping reaches an equilibrium at just under 10^−5^ s when *T* = 363–423 K. Even at *T* = 300 K, only 10^−3^ s is required for equilibration. Hence, the H-trapping/de-trapping process should always be in its equilibrium state for the load frequency range in the present experiments using 0.01–1 Hz (*i.e*., *t* ≈10° − 10^2^ s). As plotted in Figure 1(b), *θ*_T,eq_ is significantly influenced by H_2_ gas pressure. Higher temperatures amplify this pressure dependence. Conversely, the temperature dependence of *θ*_T,eq_ is weakened at higher gas pressures. The values of *θ*_T,eq_ at *T* = 300 and 423 K were 0.53 and 0.12, respectively, at *p*_H_ = 0.7 MPa.

**Figure 2.**
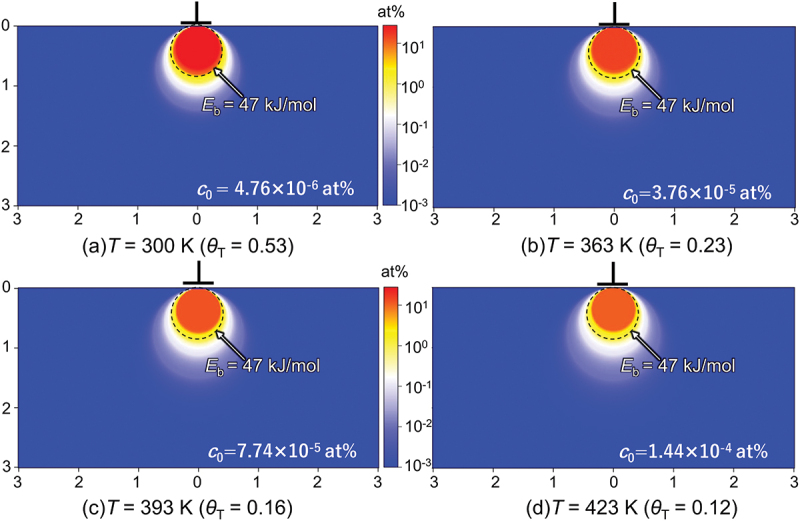
Contour maps of local equilibrium H concentration (at%) around the dislocation core for (a) *T* = 300 K, (b) *T* = 363 K, (b) *T* = 393 K, and (b) *T* = 423 K under *p*_H_ = 0.7 MPa calculated by Equation (8) and (9). The areas surrounded by dashed circles represents the regions where the binding energy with H is exceeding 47 kJ/mol.

Figure 2 shows contour maps of the local H concentration around a dislocation core, *c*, (cf. Equation (8)) for *T* = 300–423 K with *p*_H_ = 0.7 MPa. In the region close to the dislocation core with *E*_*b*_ ≈47 kJ/mol (surrounded by dashed circles), the local H concentration reaches 58 at% at 300 K but decreases with rising temperature, falling to 13 at% at 423 K. These values are two orders of magnitude greater than the average concentrations at each temperature. Notably, the calculation demonstrates the formation of a dense H-atmosphere close to the dislocation core. This atmosphere can exert a drag resistance and pinning force on the dislocation motion: this will be discussed later as a vital element of HAFCG.

### 4.2. Macroscopic properties of HAFCG

**Figure 3.**
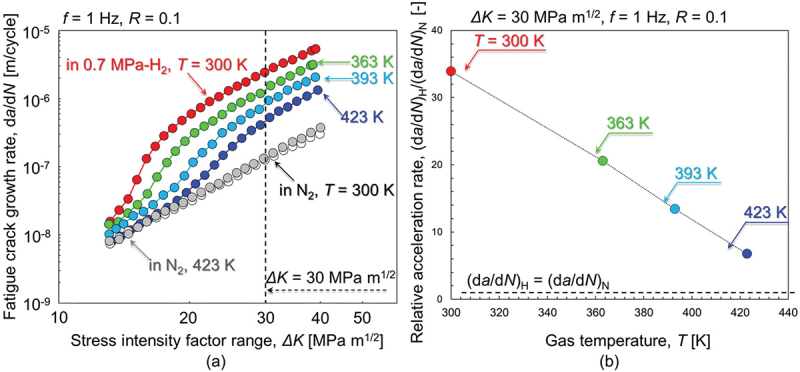
Macroscopic FCG properties in Δ*K-increasing* tests under various H_2_ and N_2_ gas environments at *f* = 1 Hz. (a) FCG rate, d*a*/d*N*, vs. stress intensity factor range, Δ*K*. (b) Relative FCG rate, (d*a*/d*N*)_H_/(d*a*/d*N*)_N_, as a function of temperature at Δ*K* = 30 MPa∙m^1/2^.

Figure 3(a) presents the FCG rate, d*a*/d*N*, as a function of Δ*K* obtained by *ΔK-increasing* tests with *f* = 1 Hz in 0.7 MPa H_2_ and N_2_ gas at *T* = 300, 363, 393, and 423 K. First, by comparing the dataset obtained in N_2_ gas at *T* = 300 and 423 K, this temperature range had no meaningful effect on the FCG rate in the absence of H. In H_2_ gas, meanwhile, there was a clear temperature effect: the higher the temperature, the lower the extent of HAFCG. The d*a*/d*N*-Δ*K* plots in H_2_ gas exhibited two-stage acceleration, resembling the propensity identified in our previous experiments [12,15,24,35]: minor FCG acceleration at a low Δ*K*, and significant acceleration at higher Δ*K*. These two regimes were defined in past studies as Stages I and II [11,12,35], the latter of which is of primary interest in the present paper. The transitional Δ*K* between these Stages I and II, Δ*K*^T^, shifted to higher values with rising temperature (*e.g*., Δ*K*^T^ = 14 MPa∙m^1/2^ at *T* = 300 K, and 20 MPa∙m^1/2^ at *T* = 423 K). To illustrate the FCG acceleration in Stage II more explicitly, the relative FCG rate in H_2_ gas with respect to that in N_2_ gas, (d*a*/d*N*)_H_/(d*a*/d*N*)_N_, at Δ*K* = 30 MPa∙m^1/2^ is shown in Figure 3(b). The (d*a*/d*N*)_H_/(d*a*/d*N*)_N_, which is a direct measure of the magnitude of HAFCG, fell as a linear function of rising temperature. For instance, (d*a*/d*N*)_H_/(d*a*/d*N*)_N_ = 33.9 at *T* = 300 K and 6.7 at 423 K.

**Figure 4.**
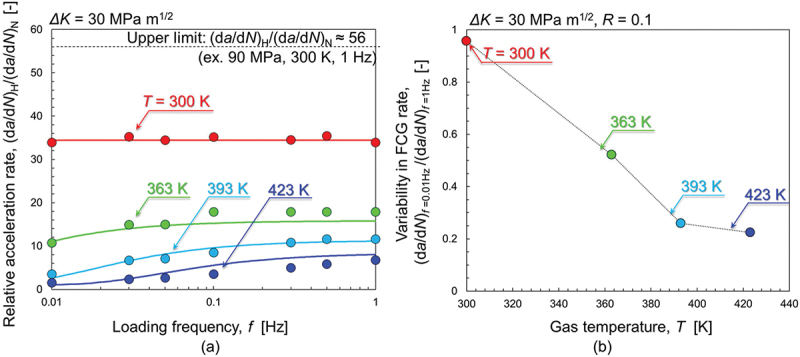
Load frequency-dependence of HAFCG in 0.7 MPa H_2_ gas at Δ*K* = 30 MPa∙m^1/2^ for various temperatures (acquired by Δ*K-constant* tests). (a) Relative FCG acceleration rate, (d*a*/d*N*)_H_/(d*a*/d*N*)_N_, vs *f*. (b) Variability of FCG rate in H_2_ gas vs temperature.

Figure 4 presents the *f*-dependence of HAFCG in 0.7 MPa H_2_ gas and its variability with temperature, a result which is of key importance in the present study. The evaluation parameter used here was (d*a*/d*N*)_H_/(d*a*/d*N*)_N_ at Δ*K* = 30 MPa∙m^1/2^ for Figure 4(a), while we used the variability of the FCG rate, (d*a*/d*N*)_*f* = 0.01 Hz_/(d*a*/d*N*)_*f* = 1 Hz_ (*i.e*., the FCG rate in H_2_ gas at *f* = 0.01 Hz divided by that at *f* = 1 Hz) in Figure 4(b). Note that the solid lines in Figure 4(a) represent the data-fitting results using Equation (12): this will be explained in Section 5 below. The *f*-dependence of the FCG rate consistently manifested in H_2_ gas except for *T* = 300 K. At a fixed temperature, the HAFCG became less pronounced with decreased load frequency. The relationship (d*a*/d*N*)_*f* = 0.01 Hz_/(d*a*/d*N*)*f* = 1 Hz is apparently a stronger function of temperature at *T* = 300–393 K than at 393–423 K. Meanwhile, it was close to being stable at unity at *T* = 300 K (*i.e*., (d*a*/d*N*)_*f* = 0.01 Hz_/(d*a*/d*N*)_*f* = 1 Hz_ ≈1.0). In Figure 4(b), one can state that (d*a*/d*N*)_*f* = 0.01 Hz_/(d*a*/d*N*)_*f* = 1 Hz_ eventually reaches a plateau beyond *T* = 393 K, which seems to be a consequence of the thermally activatable character of H-dislocation interactions (*i.e*., *T* as the denominator within the exponential terms in Equation (5) and Equation (11), representing the breakaway event of dislocation from its H atmosphere as will be discussed later in Section 5.2.3). In turn, the constancy of (d*a*/d*N*)_*f* = 0.01 Hz_/(d*a*/d*N*)_*f* = 1 Hz_ at 300 K indicates that the extent of H-dislocation interactions is invariant, or that variations in it do not cause any variation in the FCG acceleration rate at this temperature in the load frequency range of *f* = 0.01–1 Hz.

### 4.3. Fracture surface morphologies

**Figure 5.**
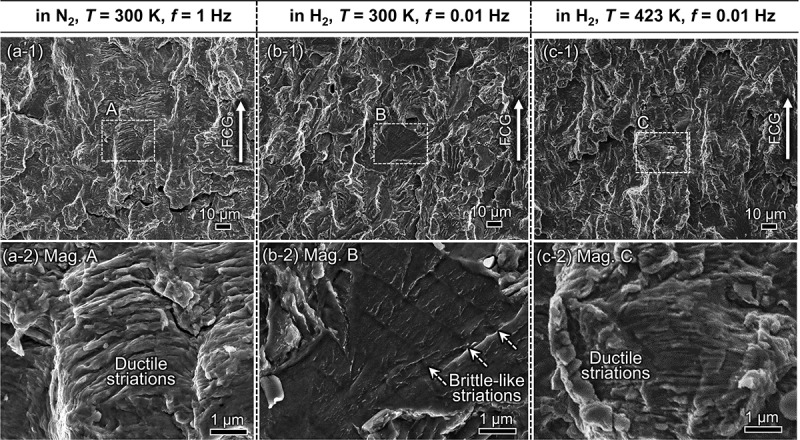
Fracture surfaces at Δ*K* = 30 MPa∙m^1/2^: (a) in 0.7 MPa N_2_ at *T* = 300 K and *f* = 1 Hz; (b) 0.7 MPa H_2_ at *T* = 300 K and *f* = 0.01 Hz; (c) in 0.7 MPa H_2_ at *T* = 423 K and *f* = 0.01 hz. The crack growth direction running from bottom to top in each image. The three images on the lower half magnify the regions marked A, B, and C in the upper half images.

Figure 5 showcases the fracture surfaces at Δ*K* = 30 MPa∙m^1/2^ in three selected test environments: (a) N_2_ gas at *T* = 300 K and *f* = 1 Hz; (b) H_2_ gas at *T* = 300 K and *f* = 0.01 Hz; (c) H_2_ gas at *T* = 423 K and *f* = 0.01 Hz. In N_2_ gas, wavy ductile striations running perpendicular to the FCG direction covered the entire fracture surface. These ductile striations are usually formed by intensive crack tip blunting and re-sharpening (in other words, crack opening-closing) processes that accompany extensive dislocation gliding along the maximum shear stress directions inside the crack tip plastic zone (CPZ) [54,55]. The average spacing of these periodic striations was equivalent to the global FCG rate.

As shown in Figure 5(b), the fracture surface was wholly replaced by a brittle appearance accompanied by grain size-scale planar facets in H_2_ gas at 300 K. Of interest is that two patterns were superposed on these facets: widely spaced brittle-like striations, and river-like serrated markings that were aligned perpendicularly to each other. This type of fracture surface is called quasi-cleavage (QC), commonly recognized as a typical fractographic characteristic of H embrittlement in low- to medium-strength BCC steels [56–59]. The spacings of the brittle-like striations also corresponded closely to the macroscopic FCG rate in H_2_ gas, as seen with N_2_, despite the substantial changes in the fracture mode caused by H. The presence of striations is a strong indication of a cycle-by-cycle fracture event, meaning that the growth of a crack is not a direct function of the crack’s opening time. This surmise is substantiated by the stable FCG behavior at *T* = 300 K (cf. Figure 4(a)) even when the load frequency is reduced.

The fracture mode at an elevated temperature (*e.g*., *T* = 423 K) at *f* = 0.01 Hz was almost identical to that in N_2_ (Figure 5(c)). One can therefore postulate that the substantial FCG acceleration seen with H_2_ gas at *T* = 300 K stems from a dramatic change in fracture morphology: raising the temperature and reducing the loading rate restores the original fracture mode in N_2_, giving rise to a mitigation of FCG acceleration.

### 4.4. Crystallographic crack propagation pathways

**Figure 6.**
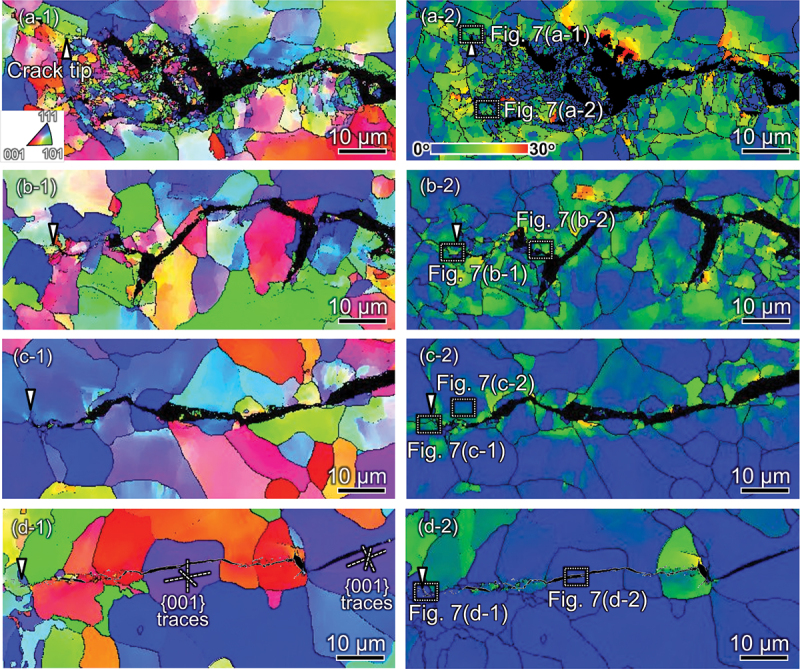
Crack propagation pathways under Δ*K* = 30 MPa∙m^1/2^: IPF maps (left side) and GROD maps (right side) analyzed by EBSD. (a) in N_2_ at 300 K, *f* = 1 Hz; (b) in H_2_ at 423 K, *f* = 0.01 Hz; (c) in H_2_ at 423 K, *f* = 1 Hz; and (d) in H_2_ at 300 K, *f* = 0.01 Hz. The ECCI micrographs of the regions surrounded by dashed rectangles are shown in Figure 7.

Figure 6 consists of inverse pole figure (IPF) maps (Figure 6(a-1) - (d-1)) acquired by EBSD analyses together with the corresponding GROD maps (Figure 6(a-2) - (d-2)) around the crack propagation pathways. The maps were acquired at the mid-thickness sections of the CT specimens, where a plane-strain state prevails. The analyzed specimens were subject to Δ*K-constant* tests at Δ*K* = 30 MPa∙m^1/2^ under the following conditions: N_2_ at *T* = 300 K; 0.7 MPa H_2_ at *T* = 423 K and *f* = 0.01 Hz ((d*a*/d*N*)_H_/(d*a*/d*N*)_N_ = 1.5); 0.7 MPa H_2_ at *T* = 423K and *f* = 1 Hz ((d*a*/d*N*)_H_/(d*a*/d*N*)_N_ = 6.7); 0.7 MPa H_2_ at *T* = 300 K, and *f* = 0.01 Hz ((d*a*/d*N*)_H_/(d*a*/d*N*)_N_ = 33.9). In N_2_ gas (Figure 6 (a-1) and (a-2)), the crack shape was wavy and frequently blanched, exhibiting totally non-crystallographic propagation. The grains located in the proximity of the crack tip or in the crack-wake were finely subdivided into small domains with different IPF colors, wherein neighboring domains show considerable crystal misorientations. This highly evolved state of intragranular substructure suggests an accumulation of cyclic plastic strain inside the CPZ prior to the passage of the crack tip.

Even in H_2_ gas, high GROD values of 20–30° were detected around the crack at *T* = 423 K (Figure 6(b-2))(*i.e*., the situation with (d*a*/d*N*)_H_/(d*a*/d*N*)_N_ of merely 1.5 (Figure 4)), accounting for an intense plasticity remaining inside the CPZ, analogous to that for N_2_. However, comparing *f* = 0.01 Hz (Figure 6(b-1) and (b-2)) and 1 Hz (Figure 6(c-1) and (c-2)) at an identical temperature of 423 K shows the crack branching to be less pronounced, the intensity of the crack-wake plasticity to be weakened, and the extension of the high-GROD zone to shrink as the load frequency is increased from 0.01 Hz to 1 Hz. This trend became more evident as the temperature decreased to 300 K (Figure 6(d-1) and (d-2)). The crack occasionally penetrated straight through several grains. These straight portions were two-dimensionally identified along {001} crystallographic planes as depicted by the plane traces in Figure 6(d-1). It should be emphasized that these straight crack pathways consistently experienced very low GROD, *i.e*., no evolution of crystal misorientation, even in extreme proximity to the crack. This has been discovered as a *less plasticity* characteristic of HAFCG, resembling the propensity confirmed in past studies [12,15,60]. When the crack encountered a grain boundary and could then be temporarily arrested, plasticity presumably developed in the adjoining grain ahead of the crack tip over a few load cycles until the crack resumed its growth. Alternatively, when the preferential crystallographic planes for cracking (mainly {001}) were unfavorably oriented with respect to the principal stress axis, the crack could be forced to propagate in a zig-zag manner, also accompanied by more or less plastic activity. These minute zones of plasticity during FCG might leave some portions of slightly high GRODs, as represented in Figure 6(d-2).

### 4.5. Dislocation substructures around the crack tip and pathways

**Figure 7.**
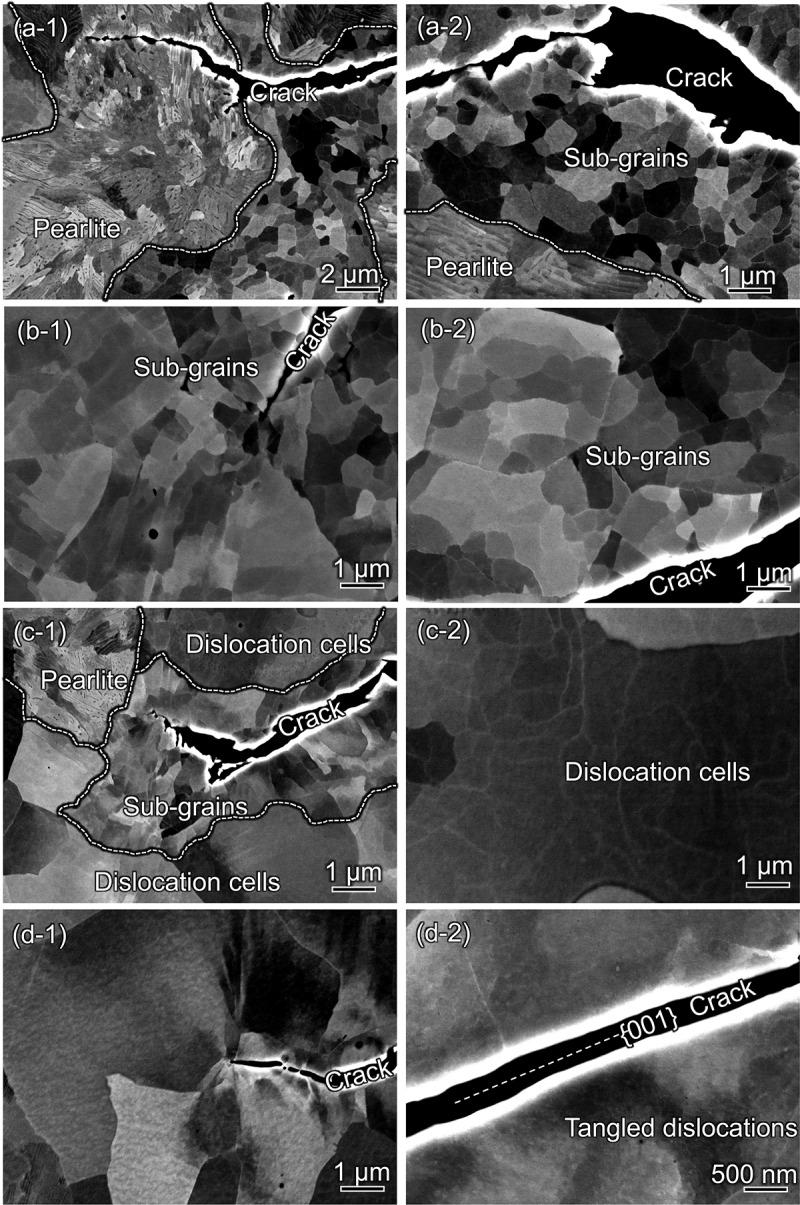
ECC images at the vicinity of crack tip or wakes subject to at Δ*K* = 30 MPa∙m^1/2^ in (a) N_2_ gas at *T* = 300 K, *f* = 1 Hz, (b) H_2_ gas at *T* = 423 K, *f* = 0.01 Hz, (c) H_2_ gas at *T* = 423 K, *f* = 1 Hz, and (d) H_2_ gas at *T* = 300 K, *f* = 0.01 Hz. The regions of interest are shown in Figure 6 (b-1) - (d-1).

Figure 7 shows the results of ECC imaging of more local regions adjacent to the cracks, giving more detail on the changes in plasticity development level caused by H, temperature, and load frequency. The corresponding regions are surrounded by dashed rectangles in the GROD maps (Figure 6, right). As shown in Figure 7(a-1) and (a-2), the dislocations in ferrite grains were well organized and arranged into sharp dislocation boundaries in N_2_. One can find fine sub-grains with diameters of less than 1 μm, each of which shows clear black-and-white contrast with respect to its surroundings. This type of structure covered whole regions around the crack except for the pearlite grains. In Figure 7(a-1), the sub-grains are positioned 10 μm distant from the crack, indicating that the crack propagated accompanied by intense repetition of plastic straining inside the CPZ. A similar feature also emerged in H_2_ gas at high temperature (*T* = 423 K) and low load frequency (*f* = 0.01 Hz) (Figure 7(b-1) and (b-2)). The presence of sub-grains confirms that the FCG event consistently involved crack tip plasticity, even in H_2_ gas, at an elevated temperature and low load frequency. This notable evolution of dislocation structures might require significant energy dissipation for the crack to propagate forward, a presumption that can be correlated with the low FCG acceleration rate of a very low (d*a*/d*N*)_H_/(d*a*/d*N*)_N_ = 1.5 (Figure 4).

Interestingly, an increase in load frequency from 0.01 Hz to 1 Hz at 423 K shrank the extent of the sub-grain-dominated zone to 1–2 μm from the crack (Figure 7(c)). Although the region relatively distant from the crack did not evolve into sub-grains, dislocation cells with small misorientations are seen in Figure 7(c-2). This shrinkage of the severe plasticity region led to the moderate HAFCG of (d*a*/d*N*)_H_/(d*a*/d*N*)_N_ = 6.7 (Figure 4).

These features were completely different with H_2_ gas at *T* = 300 K, where only weakly-evolved dislocation structures were observed, even at extreme proximity to the crack (Figure 7(d)). Adjacent to the straight crack pathways along the {001} planes with lowest GRODs (Figure 6(d-2)), only tangled dislocations or weakly-organized dislocation cells with blurred boundaries were observed. It is therefore possible to surmise that the crack tip shape kept its sharpness during the fracture, and that crack tip advancement per load cycle occurred before the onset of crack blunting. This likely triggered the significant change in dislocation substructure and, in turn, the catastrophic HAFCG of (d*a*/d*N*)_H_/(d*a*/d*N*)_N_ = 33.9. Ultimately, the evolution level of dislocation structures can be a direct reflection of the magnitude of FCG acceleration in the presence of H.

## 5. Discussion

Our systematic survey has uncovered a broader and more detailed picture of temperature- and load frequency-dependent HAFCG in a ferrite-based low-carbon steel. The primary findings were, in brief:

1. *Less plasticity* around the crack-wake in response to FCG acceleration
2. Mitigation of HAFCG by both increasing *T* and lowering *f*
3. Fracture along the {001} cleavage planes in association with accelerated FCG

In Section 5, the origin of (1) is determined first, with a brief description of the potential interactions between H and dislocations. We then attempt to formulate a model that can account for the overall phenomenology of HAFCG.

### 5.1. Plasticity development around the crack path

The plasticity evolution process inside the CPZ has been extensively studied in a variety of metals and alloys [61–65]. In general, the dislocation arrangement at a given location ahead of the crack tip transitions from discrete dislocation lines into more complex features such as tangles, walls, and cells [65]. Finally, as the crack tip approaches the location, and the applied plastic strain cycles accordingly accumulate, well-organized sub-grains are formed [61,64,65]. On the other hand, the presence of H narrowed the extent of such severely deformed zones (Figure 6(c-2)), as well as significantly restricting the evolution level of plasticity (Figure 6(d-2) and Figure 7(d)). *Are these changes an inherent impact of H on crack tip deformation character or a superficial effect stemming from other reasons?*

#### 5.1.1. Previous model and the effects of H on dislocation mobility

Murakami and Matsuoka established a *successive crack propagation* model based on the well-accepted theory of H-enhanced *localized plasticity* (HELP) [30,37]. The segregated H presumably enhances dislocation activity in the local crack tip zone and narrows the size of the CPZ, making the crack propagate by preserving its sharpness and preventing extensive blunting. The enhanced dislocation mobility by H is rooted in the *dislocation-shielding* concept, which assumes that the H atmosphere in the stress field of a dislocation reduces its repulsive interaction with surrounding obstacles [66,67]. The energy barrier against the dislocation motion is consequently reduced. This is combined with a steep concentration gradient of H from the crack tip toward the material interior. That is, the effect of increasing dislocation mobility is maximized at the crack tip, where the H concentration is the greatest, rendering the plastic activity and fracture event locally intense [37,68]. In terms of the localized plasticity model, an attempt has also been made to rationalize the peculiar mitigation of HAFCG at low loading frequencies at room temperature (Figure 4) with further consideration on the crack-tip H distribution [24]. A broadening of H distribution with a slowdown of loading rate (*i.e*., allowable time for H diffusion) expand the crack tip material volume wherein dislocation mobility is enhanced, relaxing the severity of H-induced plasticity localization. This can also seemingly be a plausible explanation for the absence of FCG acceleration at elevated temperatures because an enhanced H diffusion makes the distribution of H further broader. Note, however, that the H diffusivity in ferrite is fast enough even at room temperature (*i.e*., 10^−10^ ~ 10^−8^ m^2^/s [69,70]) so that the migration of H through the lattice takes place by a distance of 10^−5^ ~ 10^−4^ m or greater within 1 s. In contrast, the FCG rate in H_2_ gas is no more than an order of 10^−6^ m/cycle. Thus, when the FCG test is running in a steady state even at 1 Hz and room temperature, the H distribution around the crack tip should already be sufficiently broad compared with the local crack tip volume relevant to fracture.

An opposite but more straightforward idea was later proposed: H in iron conversely inhibits dislocation motion [39,71–73]. A pioneering insight gained by Song and Curtin demonstrated that the H atmosphere around an edge dislocation obstructs its motion due to being dragged behind, providing no dislocation-shielding [73]. In an H atmosphere, most of the H atoms are located very close to the dislocation core [51,73], and as a result, they may only slightly affect a stress-field of dislocation that has a long-range character. Matsumoto *et al*. performed a molecular dynamics (MD) simulation for H-dislocation interaction in iron. Their quantification also detected the significance of H-induced drag resistance for edge dislocation motion (*i.e*., shear stress for a coordinative motion with H atmosphere) with an excess of a few hundred MPa [39]. Aside from these effects, H atoms trapped along screw dislocations can facilitate the nucleation of a kink-pair [74,75]. These H atoms, however, retard the sideward motion of individual kinks [76] as computed by Itakura *et al*. [75]. Notably, the potential for suppressed dislocation mobility has been invoked for crack tip deformation in an *in-situ* experiment using a notched cantilever of Fe-Al intermetallic alloy [40].

Of these two opposing hypotheses of the enhancement or inhibition of dislocation mobility, our finding of *less plasticity* (*i.e*., an absent evidence of significant dislocation activity) seems to coincide with the latter. Evolved dislocation structures would locally be found close to the crack if the fracture actually involved a *localized plasticity* event, which was not the case in Figures 6 and 7. Nevertheless, even if the dislocations in extreme crack tip proximity are significantly affected by highly condensed H, it is questionable that such a mobility reduction can occur at a relatively long distance from the crack tip (*i.e*., the volume close to the outer circumference of CPZ), where H concentration should be quite low. The size scale of the region where reduced plasticity was observed in Figure 6 seems much larger than the zone of potential H condensation ahead of the crack tip. Thus, it is likely that *less plasticity is not an intrinsic H-impact on the dislocation mobility, but instead a superficial effect stemming from some other fundamental phenomenon.*

#### 5.1.2. Interpretation of the less plasticity problem

The lesser development of crack-wake plasticity under accelerated FCG can be more simply rationalized taking into account any H-effect on plasticity. Here, one has to imagine the steady-state propagation of a fatigue crack: it penetrates sluggishly through the CPZ, which is already present ahead of the present crack tip position. The microstructural development during this process is left behind in the crack-wake, while a new CPZ is successively formed in front of the prior CPZ location.

The CPZ size, *r*_c_, is estimated as a function of yield stress, *σ*_y_ (*i.e*., 360 MPa), and the maximum stress intensity factor, *K*_max_ (at Δ*K* = 30 MPa∙m^1/2^, *K*_max_ = 33 MPa∙m^1/2^) [77]:

(10) $$r_{c}=\frac{1}{5.6\pi }{\left(\frac{K_{\max}}{2\sigma_{y}}\right)}^{2}$$

In the present situation, the *r*_c_ at Δ*K* = 30 MPa∙m^1/2^ is 1.2 × 10^−4^ m. Thus, in N_2_ with d*a*/d*N* = 7.8 × 10^−8^ m, at least 10^3^ cycles are required for the crack tip to pass through the CPZ. During this process, the material volume inside the CPZ is subjected to severe cyclic straining, accompanied by structural evolution from randomly scattered dislocations to organized configurations of walls, cells, and sub-grains.

At a faster FCG rate, the crack must pass through the CPZ with a smaller number of load cycles, which is exactly the same as with H_2_ gas at *T* = 300 K, in which (d*a*/d*N*)_H_/(d*a*/d*N*)_N_ was 33.9 (Figure 4(a)). This change in FCG rate inherently reduces the cumulative plastic strain inside the CPZ by more than an order of magnitude, making the dislocations in crack-wake less evolved. In short, whatever the underlying mechanisms, the acceleration of FCG at a given Δ*K* (*i.e*., fixed CPZ size) weakens the dislocation structure development around the crack as an inevitable consequence. There should be a one-to-one correspondence between the magnitude of FCG acceleration and the plastic strain accumulation in the CPZ, as evidenced by the recovery of the deformation substructure at elevated temperatures or under lower load frequency conditions.

### 5.2. Roles of H-dislocation interactions

#### 5.2.1. Key presumptions and fracture model

**Figure 8.**
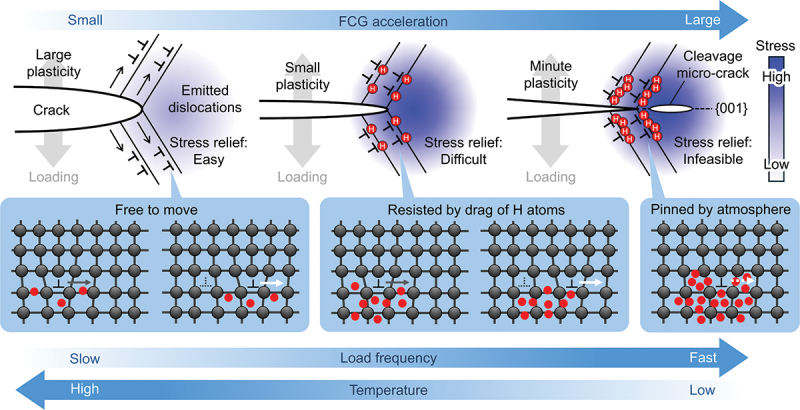
Comprehensive model of temperature- and load frequency-dependent HAFCG in H_2_ gas, based on the concept of dislocation-obstruction effects by segregated H atoms around dislocations. The primary obstruction effect is rooted from dislocation pinning by an H atmosphere at low temperature, while it is replaced by drag of H atoms behind dislocations with increasing temperature and lowering load frequency.

In line with the recent claims listed above, we put the main focus of our HAFCG model on *the decrease in dislocation mobility in the presence of trapped H*. In brief, our modeling framework is as follows. Corresponding schematics are shown in Figure 8:

1. H atoms segregate into the crack tip and are trapped by dislocation.
2. Trapped H atoms are dragged behind the dislocation, obstructing or pinning it.
3. Relief of stress concentration via dislocation motion and crack blunting is suppressed.
4. The difficulty of stress relief encompasses quasi-brittle crack propagation per load cycle, forming widely spaced striations on {001} cleavage planes.
5. Faster crack propagation reduces the plasticity development level in the CPZ.

At higher temperatures and/or lower load frequencies, the H-induced drag resistance or pinning force can be weakened due to diluted H-trapping and the dislocations’ overcoming of H via thermal activation. This weakened resistance revives the crack’s capability to plastically relax by activating dislocations that regain their mobility. In our conclusion and summary of this paper below, the model is substantiated by our experimental findings, followed by a theoretical formulation that successfully fits FCG behavior in practice.

#### 5.2.2. Dislocation drag of core H atoms

Concerning the stability of H-trapping by dislocations, only an equilibrium state must be considered (Figure 1(a)). The local H concentration, *c*, around the dislocation core under a given binding energy, *E*_b_, is directly correlated with *θ*_T,eq_ (Equation (5)). Taking *E*_b_ to be 47 kJ/mol [51], a temperature elevation from *T* = 300 to 423 K eventually reduces *θ*_T,eq_ from 0.53 to 0.12 and thereby decreases *c* from 58 at% to 13 at% (Figure 1(a) and Figure 2), which clearly dilutes the H concentration in the atmosphere. Lu *et al*. report that the maximum binding energy of H with a dislocation core is 0.42 eV (≈40.5 kJ/mol), and these trapped H atoms can boil off when elevating the temperature from 300 to 600 K [78]. At these elevated temperatures, where diffusion of H atoms is sufficiently fast, their possible obstruction mode for dislocations is most likely solute drag. However, in the MD simulations performed by Matsumoto *et al*. [39], the shear stress for dragging H with an edge dislocation is 433 MPa at *T* = 300 K, 100 MPa at *T* = 400 K, and 8 MPa at *T* = 500 K at a dislocation velocity of 0.01 m/s. Thus, although drag resistance can be more or less present, it is ineffective at elevated temperatures and low dislocation velocities because of a diluted H atmosphere and the high followability of the H atoms themselves. As such, FCG acceleration should not manifest: a typical example is *T* = 423 K with *f* = 0.01 Hz where (d*a*/d*N*)_H_/(d*a*/d*N*)_N_ is only 1.5. Meanwhile, at a faster dislocation velocity of 0.1 m/s at *T* = 400 K, the MD simulation [39] indicated a drag stress of ≈ 400 MPa. This suggests that, in terms of our fracture model, the FCG acceleration can emerge even at higher temperatures if the loading is fast. This estimation coincides qualitatively with the meaningful rise of (d*a*/d*N*)_H_/(d*a*/d*N*)_N_ to 6.7 at *T* = 423 K after fastening the load frequency at 423 K. The FCG acceleration rate was greatly decreased from 6.7 to 1.5 by reducing the load frequency from 1 to 0.01 Hz. The competitiveness between dislocation motion and H followability, as well as the local concentration of H in the atmosphere, controls this temperature- and load-frequency-dependent HAFCG characteristics at relatively high temperatures.

#### 5.2.3. Dislocation pinning and breakaway

Given that H around the dislocation core acts as an obstacle to dislocation motion [39,73], the pinning of dislocations by their H-atmosphere is also envisaged, a breakaway from which can be regarded as a thermally activated event. This is the case, for instance, at *T* = 300 K, where the diffusivity of H is relatively low, and the H concentration in the atmosphere is high. It also applies to slightly higher temperatures with high load frequency where allowable time is short and reduces the probability of breakaway. The pinning of dislocations at the crack tip can also trigger FCG acceleration in accordance with the presently proposed model.

The probability of a thermally activated breakaway of this type from the solute atmosphere, *P*_break_, has been formulated by Lothe [79] following an Arrhenius-type rate equation:

(11) $$P_{\mathrm{break}}\begin{array}{c} =\exp\left(-\frac{E_{c}}{\mathrm{RT}}\right) \end{array}$$

where *E*_c_ is the activation energy for a thermally activated breakaway. This parameter can be expressed as a function of *θ*_T,eq_ by multiplying the H concentration-dependent constant, *C*_1_, and the binding energy of H (*i.e*., *E*_c_ = *C*_1_*θ*_T,eq_*E*_b_). The value of *P*_break_ fluctuates between 0–1, being assumed here as the chief control parameter for the extent of HAFCG.

In turn, the stability of dislocations to remain pinned by the H-atmosphere during a time *t* is given by (1−*P*_break_)^*t*^. Ultimately, the FCG rate in H_2_ gas at a given temperature and load frequency, (d*a*/d*N*)_H_, can be expressed as follows in its simplest form:

(12) $${\left(\frac{\mathrm{da}}{\mathrm{dN}}\right)}_{H}={\left(1-P_{\mathrm{break}}\right)}^{C_{2}\;/\;f}\left\{{\left(\frac{\mathrm{da}}{\mathrm{dN}}\right)}_{H,\max}-{\left(\frac{\mathrm{da}}{\mathrm{dN}}\right)}_{N}\right\}+{\left(\frac{\mathrm{da}}{\mathrm{dN}}\right)}_{N}$$

where (d*a*/d*N*)_H,max_ is the maximum FCG rate in H_2_ gas, the upper limit of FCG acceleration, and *C*_2_ is a fitting constant connecting *t* with the load frequency. Here, (d*a*/d*N*)_H,max_ is defined as 4.34 × 10^−6^ m/cycle, which is the FCG rate in a more extreme H_2_ gas environment (*p*_H_ = 90 MPa, *T* = 300 K) at Δ*K* = 30 MPa∙m^1/2^ and *f* = 1 Hz [24,29]. Under harsh conditions such as this, *θ*_T,eq_ approaches unity.

The experimental results at various temperatures and load frequencies were fitted with Eq. (12), and the fitting curves are drawn in Figure 4(a) with the solid lines. At all the examined temperatures, good correspondences between the FCG acceleration behavior and Equation (12) were evident under the two fitting constants of *C*_1_ = 1.20 and *C*_2_ = 0.28. Note that the use of Equation (12) to fit the data at high temperatures appears incompatible with the assumption of drag resistance in Section 5.2.2. Nonetheless, drag motion requires successive short-range jumps of individual H atoms in the direction of the dislocation movement, which can also be regarded as a framework for breakaway events on an atomic scale. The feasibility of this type of motion is also expressed in Arrhenius form, as in Equation (11) [39,80], including the binding energy of H and the H concentration along the dislocation line in the numerator of the exponent. Thus, the universality of Equations (11) and (12) can express both forms of dislocation motion via solute drag and breakaway.

The success of Equation (12) in fitting the practical data substantiates our concept of HAFCG predicated on chiefly the responsibility of H-induced obstruction for dislocation motion at the crack tip. In real experiments, the drag resistance and pinning force compete mutually, the latter of which may become increasingly important and lead to more substantial FCG acceleration at a lower temperature and higher load frequency. In summary, our new model (schematically illustrated in Figure 8) in Section 5.2.1 explains the overall behavior of HAFCG from both perspectives, *i.e*., its dependencies on experimental variables and microscopic crack propagation pathways. Note also that our present discussion framework is built upon the presumption of equilibrium H distribution in the local crack tip zone relevant to fracture (Section 4.1). If the loading frequency is too high or the temperature is too low so that the state deviates from equilibrium, the kinetics of H absorption and diffusion may also be taken into account.

## 6. Summary and conclusions

The temperature- and load frequency-dependent hydrogen-assisted fatigue crack growth (HAFCG) of ferrite-based low-carbon steel was investigated in 0.7 MPa H_2_ gas. Combined with the fractographic observations and the analysis of crack propagation pathways, a new model that rationalizes the overall characteristics of HAFCG was established using a successful theoretical formulation. The crack propagation behaviors under the presently employed conditions are finally summarized using classification by local H concentration in the atmosphere around a dislocation.

1. *High H concentration* (*e.g., T* = 300 K)
  The dislocations emitted from the crack tip are pinned and suddenly immobilized by densely-segregated H. The crack propagates in a quasi-brittle manner without blunting, preferentially passing through {001} cleavage planes. The acceleration of FCG is substantial with the (d*a*/d*N*)_H_/(d*a*/d*N*)_N_ beyond 30. Thermal activation is not feasible for triggering a breakaway of dislocation from the H atmosphere on a realistic time scale, thus giving rise to FCG acceleration that is mostly independent of the load frequency. Because of the fast FCG rate itself, only weakly-evolved deformation substructures are left behind the crack tip.
2. *Moderate H concentration* (*e.g., T* = 369–393 K)
  Pinning is more or less reduced due to the diluted H atmosphere. Instead, drag of H becomes increasingly important in obstructing the dislocation movement. The crack propagates in a mixture of quasi-brittle and ductile modes, leading to a moderate HAFCG (*e.g*., (d*a*/d*N*)_H_/(d*a*/d*N*)_N_ = 10–20) accompanied by slightly deformed microstructures in the crack-wake. Unlike *T* = 300 K, load frequency is of primary importance. Lowering *f* plays a double role of facilitating thermally-activated breakaway from the atmosphere and reducing drag resistance. These increasingly attenuate FCG acceleration in the frequency range of 0.01–0.1 Hz
3. *Low H concentration* (*e.g., T* = 423 K)
  The situation is similar to the above Case 2, in which a more diluted atmosphere can exert only weak drag resistance at higher load frequencies of above 0.1 Hz. At all lower load frequencies, it is too easy for dislocations to overcome the H atoms acting as obstacles, resulting in a negligible FCG acceleration rate of (d*a*/d*N*)_H_/(d*a*/d*N*)_N_ = 1.5. Due to this FCG rate, which is almost equivalent to that seen with N_2_, the plasticity around the crack fully recovers to its well-evolved state.

## Data Availability

The raw/processed data required to reproduce these findings cannot be shared at this time, as the data also forms part of an ongoing study.
